# The Role of the Caudal Superior Parietal Lobule in Updating Hand Location in Peripheral Vision: Further Evidence from Optic Ataxia

**DOI:** 10.1371/journal.pone.0046619

**Published:** 2012-10-05

**Authors:** Joshua A. Granek, Laure Pisella, Annabelle Blangero, Yves Rossetti, Lauren E. Sergio

**Affiliations:** 1 School of Kinesiology and Health Science, Centre for Vision Research, York University, Toronto, Ontario, Canada; 2 Impact - Centre de Recherche en Neurosciences de Lyon, Inserm U 1028, CNRS UMR 5092, Bron, France; 3 Biologie Humaine, Université Lyon 1, Lyon, France; 4 Mouvement et Handicap, Hospices Civils de Lyon, Hôpital Henry Gabrielle, St-Genis-Laval, France; 5 Department of Biology, City College of New York, New York, New York, United States of America; McMaster University, Canada

## Abstract

Patients with optic ataxia (OA), who are missing the caudal portion of their superior parietal lobule (SPL), have difficulty performing visually-guided reaches towards extra-foveal targets. Such gaze and hand decoupling also occurs in commonly performed non-standard visuomotor transformations such as the use of a computer mouse. In this study, we test two unilateral OA patients in conditions of 1) a change in the physical location of the visual stimulus relative to the plane of the limb movement, 2) a cue that signals a required limb movement 180° opposite to the cued visual target location, or 3) both of these situations combined. In these non-standard visuomotor transformations, the OA deficit is not observed as the well-documented field-dependent misreach. Instead, OA patients make additional eye movements to update hand and goal location during motor execution in order to complete these slow movements. Overall, the OA patients struggled when having to guide centrifugal movements in peripheral vision, even when they were instructed from visual stimuli that could be foveated. We propose that an intact caudal SPL is crucial for any visuomotor control that involves updating ongoing hand location in space without foveating it, i.e. from peripheral vision, proprioceptive or predictive information.

## Introduction

Humans typically gaze and reach directly toward objects they interact with, a situation that has been termed “standard” [1]. In tool-use however, the direction of our gaze and the object that we are manipulating are often in different spatial locations (e.g. driving). These “non-standard” situations require the mapping between stimulus and response to be learned and calibrated [1]. Commonly performed “non-standard” situations often include the integration of various transformational (e.g. push computer mouse forward to move cursor upward) or arbitrary (e.g. green light means push gas pedal) rules. Such cognitive visuomotor associations are preserved in optic ataxia (OA) [2]–[4], suggesting that the caudal superior parietal lobule (SPL) damaged in these patients is not crucial for this ability. However, neuroimaging findings give evidence of an involvement of the posterior parietal cortex (PPC) in non-standard visuomotor mapping (see below), the nature of which is unclear. Here, we put forward that the involvement of the SPL is related to another characteristics of “non-standard” situations: they often include having to guide actions outside the field of view or in peripheral vision. Both the explicit strategic control of non-standard transformational mappings [5]–[10] and the implicit adaptation to spatial orientation differences between sensory modalities (e.g. vision and proprioception) [11], [12] imply an ability to know or predict hand location during motor execution without direct vision. The updating and sensorimotor transformation of proprioceptive information has recently been shown to be impaired in OA [13], which indicates that OA patients may need to look at their hand in such situations.

Brain imaging research has revealed overlapping yet distinct cortical networks involved in different types of non-standard reaching [14]–[17]. A common cortical region activated during non-standard reaching is the PPC, which has been established as a predominant contributor to the preparation and execution of this type of non-standard behavior. Within the PPC, the caudal portion of SPL (delimited ventrally by the intraparietal sulcus and posteriorly by the parieto-occipital sulcus), is known to be directly connected to the rostral dorsal premotor cortex (PMd) [18] and to constitute the visual dorsal stream [3]. The intraparietal sulcus and the SPL have been shown to display increased activity during visuomotor adaptation [19] and during mental rotation [20]. Similarly, greater activity within the medial superior parietal region has been observed for anti-pointing relative to pro-pointing during central fixation [14]. Alternatively, other studies have concluded from endpoint errors that anti-pointing relies on a visuo-perceptual network which can be dissociated from the direct visuomotor network which supports pro-pointing [21], [22]. Based on evidence from patients with neglect, this visuo-perceptual network could include the inferior parietal lobule [23], [24] and the superior temporal gyrus [25], since such patients with neglect (contrary to patients with unilateral OA [22]) show non-lateralised deficits of anti-saccade [26] and anti-reaching [27]. The process common to pro- and anti-pointing involving the most caudal portion of the SPL might thus be the control of a reach towards an extra-foveal position [28]. An extensive PPC network is involved even as gaze and hand direction *begin* to become decoupled (for review, see [29]). In addition, neurophysiological recordings in area V6A, a monkey medial area at the parieto-occipital junction [30], [31], have offered further evidence that neurons within the medial parieto-occipital cortex are involved in proprioceptive updating in situations in which gaze direction has been decoupled from reach direction [32].

Patients with optic ataxia (OA), which is a visuomotor disorder that is associated with damage to the caudal SPL [33], [34], present an ideal population to decipher the role that the visual dorsal stream plays within the neural network responsible for preparing and guiding different types of visually-guided reaching. With preserved primary visual and motor function, OA patients typically exhibit misreaching [35] and impaired visuomotor on-line control [36]. We have recently proposed that the deficit associated with OA is a combination of a faulty coding of extra-foveal locations in their contralesional visual field (Field effect) and a faulty proprioceptive transformation of the location of their contralesional hand for reaching in the whole space (Hand effect) [3], [4], [13], [35]. This proprioceptive transformation is necessary in conditions restricting visual feedback of the hand (as in the dark [13]) or in conditions where a provided visual feedback is decoupled from real hand location or direction. The involvement of caudal SPL in visually-guided reaching toward extra-foveal targets has been well accepted as well as the spared performance of OA patients in “standard” conditions of direct visually-guided reaching in free vision [2]–[4]. However, the question remains if caudal SPL is a crucial component in guiding a reach within peripheral visual space when one is free to foveate the target, but the limb motion is spatially decoupled from gaze direction, a skill used in everyday life.

In order to address the role of the caudal SPL in situations in which the hand location is decoupled from gaze, we investigated a series of non-standard visuomotor tasks. The participants were briefly trained to perform visuomotor tasks that required the application of both cognitive and spatial algorithms in order to align a cursor with a foveated visual target using their hand. The spatial algorithms included the manipulation of cursor feedback rotation and the spatial plane of the hand movement (performed in isolation and in combination).

The *first* aim of the present study was to test the role the dorsomedial parieto-frontal neural pathway from caudal SPL to rostral PMd [18] in performing different types of non-standard visuomotor mappings. Specifically, we predicted that an intact caudal SPL served as a crucial node for the preparation and guidance of visually-guided reaches in situations in which the hand was spatially decoupled from gaze direction. In contrast, we predicted that an intact caudal SPL was not crucial for the control of standard, spatially coupled visually-guided reach movements, nor for the control of arbitrary mappings (which also do not involve eye-hand decoupling). We therefore expect larger spatial endpoint errors or increased movement timings in patients relative to controls in the non-standard conditions, even if the subjects are free to look at the target. This deficit under conditions of eye-hand decoupling may reflect an inability to process *simultaneously* the decoupled hand and eye targets without an intact caudal SPL [37]. This inability may be explained in two ways which lead to two different predictions.

The first explanation is that the caudal SPL represents extrafoveal locations (of the hand or the goal) as we postulated previously [4], [13]. Along this positional hypothesis (developed in [38]), lateralised effects would concern the right visual target which forces the patients to monitor their hand location (from proprioception or from the cursor) in their left (contralesional) visual space. In contrast, in visuomotor rotation conditions, where the visual target location has to be intentionally remapped to its symmetrical location in the opposite visual field for anti-pointing, it is expected that only the left visual target presentation will be affected. Indeed, it is known from recent results that only targets presented in the left (contralesional) visual space will be erroneously remapped for anti-reaching [22]. Given these opposing effects, along the positional hypothesis [4], [13] we are unlikely to observe lateralised spatial effect of target presentation side.

An alternative explanation is that the key factor is neither the hand location nor the extrafoveal goal location per se, but rather their spatial relationship (allocentric coding), such that the deficit is determined by the direction of the required movement. This directional dependence could arise if the dorsal stream in each hemisphere subserves contralaterally-directed orienting behaviour (cf. [39]). According to this ‘directional’ hypothesis (also more recently developed by [38]), a unilateral optic ataxic patient with field dependent misreaching would fail when contralesionally-directed guidance is required (leftward movements in our left OA patients). In such a case, we should observe a lateralised deficit depending on the motor goal, which is opposite to the side of visual target presentation in visuomotor rotation conditions. However, other authors [40], [41] have hypothesized that this guidance based on allocentric coding relies more on the ventral visual stream system (because it is impaired in patient D.F. with visual agnosia and is processed slower than target-directed coding).

The *second* aim of the present study was to explore the “natural” eye scan path behaviour of OA patients in situations in non-standard conditions (for eye-hand coordination strategies in direct (standard) reaching conditions, see [42]). In our non-standard conditions, the decoupling of the spatial targets of the effectors was not due to extra-foveal reaching during central fixation – as done in most previous work with OA patients – but due to having the eyes and hand move to different locations in space. We predict that OA patients will not be able to simply saccade towards the target and maintain fixation during the performance of a decoupled visually-guided reach (control behavior), but will rely on additional eye movements in order to successfully complete the task (i.e. to recalibrate their hand and goal locations using central vision). The more complex the condition is, the more we may observe a tendency of the patients to make additional eye movements. Indeed, the patients may compensate their deficit by alternating several eye movements between the goal and the hand locations (either by looking at the real hand or by looking at the visual feedback cursor) in order to recalibrate visually their hand location.

## Methods

### Ethics statement

All participants signed informed consent and the study protocol was approved by the York University human participant research ethics committee, certificate number 2008-098.

### Subjects

The participants were two patients with dorsal visual stream damage (CF, male, age 30; MFL, female, age 60) and eight healthy age-matched controls (four controls - two female - per patient; mean ages 30±4 and 59±5). All participants were tested for handedness [43]. Control subjects were tested using their dominant right hand (handedness score greater than +0.50), while the patients were tested with both hands. MFL is predominantly left handed (although trained to use her right hand as a child; her handedness score was left-handed, −0.53). CF is predominantly right-handed (although his handedness score indicated ambidextrous, +0.33). CF reported to be an avid video-gamer, with a self-reported skill level of 8/10 prior to brain injury (although he reported a decrease in ability to 4/10 post-injury) and practiced 2–3 hours/week, while MFL had no video-game experience. All subjects had experience with a computer mouse and/or laptop touch pad.

### Patient details

**Figure 1:**
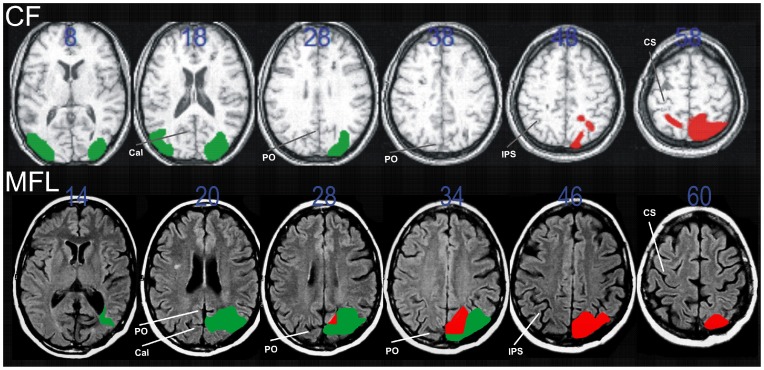
Anatomical MRI scan slices of patient CF (first row) and patient MFL (second row). The z-coordinates of the axial slices are indicated in blue. Occipital and parietal lesions were mapped and colored in green and red respectively. The major sulci are indicated to guide the localization of the lesions (Cal: calcarine, PO: parieto-occipital, IPS: intra-parietal, CS: central). Note that these MRI scans were acquired at the acute stage of the strokes and that at the time of testing no visual field defect was associated with the occipital lesions. The patients' lesions overlap to the greatest extent at the level of the right caudal superior parietal lobule, which is the pertinent anatomical substrate of their common chronic visuomotor deficits.

At the time of testing, patient CF was a 30-year-old male who suffered a watershed posterior infarct six years earlier, resulting in distributed and asymmetrical bilateral lesions of the occipito-parietal region (Brodmann's areas 18, 19, 7, 5 and 2) with a minute extension to the semiovale centers (Fig. 1 – top row). At the time of testing, most lesions were asymptomatic; he exhibited chronic unilateral left optic ataxia, thought to be the consequence of intra-parietal sulcus lesion only in the right hemisphere, as well as larger SPL and white matter damage in the right hemisphere, probably causing a parieto-frontal disconnection from intra-hemispheric fibres lesions (Fig. 1 – top row; for other behavioral details, see [36], [44]).

At the time of testing, patient MFL was a 60-year-old female who suffered from haemorrhagic stroke in the right hemisphere 16 years earlier. The lesion damaged the caudal part of the intraparietal sulcus and of the SPL (Fig. 1 – bottom row). Following this focal lesion in the right hemisphere, MFL exhibited unilateral left optic ataxia (for an example of her behaviour, see [22]).

Patients were given a set of standard clinical tests involving visual field topography (Goldman perimetry), sensory stimulation tests (visual and tactile extinction), neurological evaluation of reflexes and muscle tone and joint movements. Neither patient exhibited any purely motor, somatosensory or visual deficits, or any sign of neglect (on standard line bisection, star cancellation and drawing tasks).

### Experimental procedure

Subjects sat in front of a computer monitor (41 cm from screen), head-fixed (with a chin rest), in a darkened room, and made sliding finger movements over a touch sensitive screen (Keytec Magic Screen: Model KTMT-1315: Sampling rate: 100 Hz) from a center target (with a four second delay) to one of four peripherally presented targets (up, down, left, right). The targets were presented 95 mm (13° visual angle) from the central target and were 25 mm in diameter on the vertical monitor. Subjects were instructed to move as accurately and quickly as possible, across the touch screen and encouraged to maintain a consistent initial arm orientation for the different task conditions of the experiment. Right eye movements were monitored (Cambridge Systems, 250 Hz and EyeLink II, 250 Hz). The viewing space was calibrated using a nine-point calibration and drift correction was applied between each condition.

**Figure 2:**
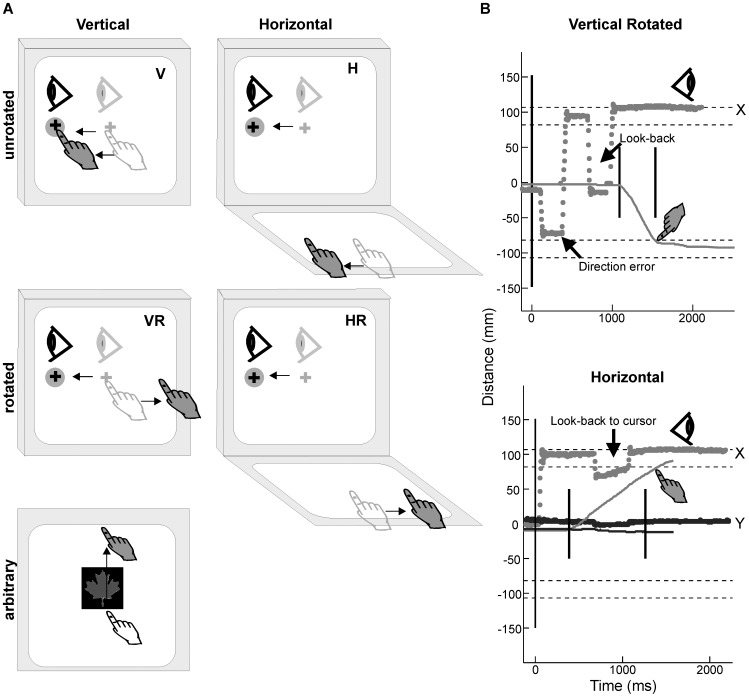
Task procedure, example patient eye data. (A) Schematic drawings of the standard center-out reaching movement towards one of four peripheral targets. Reaching movements were done both directly (vertical) and in two basic manipulations: spatial plane dissociation (horizontal) and 180° visuomotor rotation (vertical rotated) employed both separately and in combination (horizontal rotated), as well as a single arbitrary association task. In the arbitrary condition, the maple leaf symbol is shown to indicate a required upward hand and eye movement. (B) Example x (gray dots) and y (black dots) eye position (in mm) for OA patients towards right (positive x) peripheral target during the VR and H conditions. Gray lines represent x hand position and black line represents y hand position (positive is upward) from movement onset to movement offset (short black lines). Horizontal dashed lines demark the location of peripheral targets, while long vertical black lines represent the go signal. Note that the look-back in the H condition was to the cursor representation of the hand, not the hand itself, while the look-back in the VR condition was to the hand.

The subjects performed four conditions and a single arbitrary condition (Fig. 2A), each of which consisted of 20 trials. All conditions were performed in randomly assigned blocks, towards randomly presented visual targets. Initial training (up to 40 trials) was performed by all subjects prior to each condition until each subject reported that they were adequately prepared to ensure equal understanding of the task. Importantly, in order to emulate a natural environment, all subjects were instructed to look at the visual target (i.e. foveal acquisition), but were not restricted to a certain eye scan path. In the darkened room, the border of the computer monitor and the hand were still visible with peripheral vision. The subjects performed a single standard reaching task (‘Vertical’; V), in which reaching movements were performed directly on the touch screen which was placed directly over the vertically-displayed monitor, and the cursor feedback reflected veridical finger motion. Subjects also performed three non-standard transformational reaching tasks involving two basic manipulations employed both separately and in combination:

A ‘Horizontal’ (H) condition, in which reaching movements were performed on a touch screen which was placed in the horizontal plane in front of the vertically-displayed monitor, a ‘Vertical Rotated’ (VR) condition, in which reaching movements were performed on a touch screen which was placed directly over the monitor, but the cursor feedback that reflected finger motion was rotated 180°, and a ‘Horizontal Rotated’ (HR) condition, involving a combination of the two manipulations, whereby reaching movements were performed on a touch screen which was placed in the horizontal plane in front of the vertically-displayed monitor and the cursor feedback reflected finger motion that was rotated 180°.

The unilateral OA patients (MFL and CF) were tested on the standard and the three non-standard transformational reaching tasks using both hands (to explore possible hand effects). In order to assess general strategic control in each of our patients relative to the control group, a single non-standard arbitrary association reaching task (ARB) was performed by the OA patients with their contralesional limb, while the controls used their dominant limb. Briefly, the ARB condition consisted of four different symbols presented in the center of the monitor which each represented a different target location, whereby the subjects were given feedback of the target at the completion of a successful trial. The maple leaf symbol was shown to represent the top target, the Bentley™ symbol reflected the left target, the Acura™ symbol reflected the right target and the Blue Jay™ symbol reflected the bottom target.

### Data analysis

Trials were only included in the hand movement timing, path, and endpoint analyses if they were successfully performed within a maximum of eight seconds without a 180° hand direction reversal (hand path errors were enumerated in a separate analysis).

An index of difficulty (ID) for each subject using 11 dependent variables (*i*) was computed as a measure of how demanding eye-hand decoupling (NS; non-standard, our VR, H, and HR conditions) was relative to direct visuomotor control (S; standard, our Vertical condition) by using the following formula:
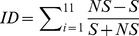



Hand movement timing was analyzed whereby hand reaction time (***RT***) began when the peripheral target was presented and ended at movement onset. Hand movement onsets were scored as the point at which the resultant of the x and y trajectories exceeded 10% of the peak velocity using a custom-written computer algorithm; the scored point was then verified visually for each trial (i.e. before any corrective movements). The hand ballistic movement time (***MT***) for all conditions began from the hand movement onset and ended at the first point in which the movement slowed to 10% peak velocity. In order to quantify the timing for corrective movements, we analyzed corrective movement time (***CMT***), which began at the end of MT (10% peak velocity) of a given trial and ended when the cursor entered the perimeter of the peripheral target (trial completion).

The individual hand movement paths were first low-pass Butterworth reverse filtered at 10 Hz (Matlab, Mathworks Inc.). Hand movement paths were recorded as direction reversals (***DR***) if the first half of the paths in each trial deviated 180° or 45° (errors classified separately) from a straight line towards the cued direction. Hand movement accuracy parameters were determined from the participant's mean movement endpoints for each target location and analyzed separately for distance errors (***on-axis CE***) and for direction errors (***off-axis CE***),. Endpoint precision (variable error, ***VE***) was determined by the distance of the endpoints of the individual movements from their mean movements.

Eye scan paths were also tested in order to observe the un-restricted eye movement behaviour when the hand was spatially decoupled from gaze direction. The eye scan paths were only analyzed for a given trial if the corresponding hand movement trial was successfully completed. Eye movement onset was determined at 10% peak saccadic velocity following central fixation. Each sampled data point obtained during the experiment that was registered as a blink was smoothed off-line using data obtained from the nearest accurate measurement before and after the point. Blinks were detected from a transient reduction in the pupil size measurement, provided by the eye tracking system. Eye scan path data were recorded from eye movement onset up until 1500 msec of peripheral target hold in order to be able to identify saccade-related errors. The saccade-related errors were placed into three categories: 1) initial direction errors (***DE***), 2) ***look-backs***, and 3) ***steps*** to catch up the target. DE were defined as initial primary saccades towards the wrong target (at least 90° away from the correct target) travelling a minimum of 50% of the distance between the central and peripheral target. Look-backs were counted when subjects reversed eye direction (towards the hand or the cursor) a minimum of 20% of the total amplitude from the central to peripheral target, holding at least 100 msec. Saccade-related errors were categorized as ‘teps’ if an eye movement was at least 10% of a full saccade from central to peripheral target, holding for at least 100 msec. Hypometric saccadic steps were defined as brief saccadic pauses occurring before reaching the peripheral target, while hypermetric steps were recorded when these small saccadic pauses occurred beyond the peripheral target location towards the boarder of the computer monitor.

### Statistics

The data from the individual patients and the controls were analyzed separately. For the control group (n = 8), we conducted two-way repeated measures ANOVAs with condition and target as within-subject factors, and age (younger group - 30±4 vs. older group - 58±5) as a between-subject factor in order to address possible age by condition interactions. All ANOVA results were reported with Greenhouse-Geisser-corrected *p*-values, and post hoc comparisons were corrected for multiple comparisons (Bonferroni).

Inter-group analyses were performed on MFL and CF separately using modified t-tests [45]; each hand separately) and were compared with the control group for each visual target, in order to screen for hand and/or visual field effects (i.e. target direction). Importantly, for accurate comparison of each case (MFL and CF), the modified t-tests utilized in the current study adjusted the critical t-value depending on the variability (i.e. standard deviation) and group size of our control group (for details, see [45]). Therefore, alpha levels for all inter-group analyses were adjusted to 5% at *p*′<0.05 [45]. In addition, an index of the number of standard deviation units that each case differed from a randomly chosen control subject (i.e. ‘effect size’) was calculated for each modified t-test to demonstrate the magnitude of the difference between groups [45]. One exception was during the comparison of the number of initial saccadic direction errors between the patients and the control group. Since the control group did not perform such errors (mean 0±0), no statistical comparison could be performed.

## Results

Because the patients were 30 years apart in age, we tested two different age-matched control groups. Importantly, no condition by age interactions were observed within the control group for any dependent variable (*p*>0.05). Therefore, all inter-group analyses were performed for each OA patient relative to the entire control group (n = 8). For details on the individual dependent variables see below.

### Index of difficulty

**Figure 3:**
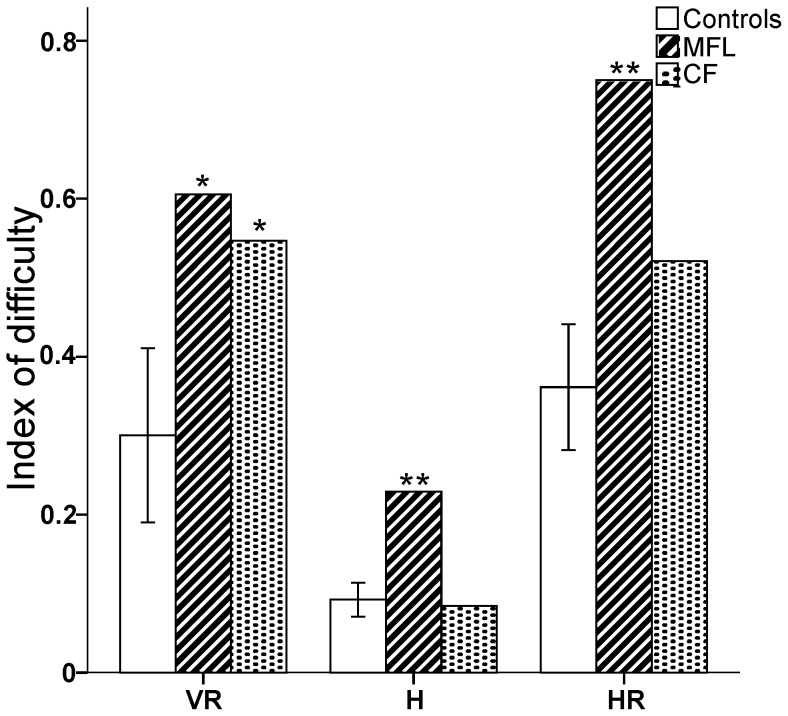
Index of difficulty for the decoupled non-standard conditions relative to the standard condition. A positive number from 0–1 indicates that the decoupled conditions were more difficult than the standard conditions across all significant dependent variables. Note the marked increase in ID for both patients in the Vertical Rotated (VR) condition relative to the controls. Error bars denote 95% Confidence Intervals. *p′<0.05 ; **p′<0.01.

We calculated an index reflecting the performance demand of the different non-standard transformational conditions relative to the standard condition (see Methods for details). For each subject, the index of difficulty (ID) was always positive, indicating that decoupling gaze and hand target location was more challenging than direct visuomotor control (Fig. 3).

#### Control group

Control subjects varied in their performance depending on the level of eye-hand decoupling (main effect of condition; ANOVA, *F*_2,11_ = 26.3, *p*<0.0001), whereby VR was more demanding than H (*p*<0.05) and HR was more demanding than both VR and H (*p*<0.05).

#### OA patients versus control group

MFL struggled in all conditions when gaze and hand position were decoupled relative to control participants (VR: *t* = 2.9, *p*′<0.05, effect size = 3.1, H: *t* = 5.1, *p*′<0.01, effect size = 5.4, HR: *t* = 3.8, *p*′<0.01, effect size = 4.1), while the index of difficulty was significantly higher than controls only in VR (*t* = 2.4, *p*′<0.05, effect size = 2.5) for CF.

### Hand movement timing

#### Control group

Within-group analyses were conducted on movement timing to determine a baseline of difficulty depending on the condition and the target. Condition main effects were observed for movement preparation (RT; ANOVA, *F*_2,14_ = 11.4, *p*<0.001), ballistic movement timing (MT; ANOVA, *F*_2,10_ = 6.2, *p*<0.05), and online movement correction (CMT; ANOVA, F_1,9_ = 4.9, *p*<0.05). Post hoc comparisons revealed longer RT for VR compared with H, and HR compared with H and V (*p*<0.05). Target direction did not influence movement timing parameters within this group (*p*>0.05).

#### OA patients versus control group

**Figure 4:**
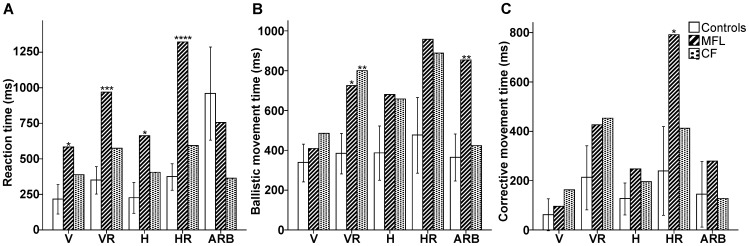
Hand movement timing data for MFL, CF, and the control group. Mean reaction times (A) ballistic movement times (B) and corrective movement times (C) in msec for both groups for the five conditions (V = Vertical; VR = Vertical Rotated; H = Horizontal; HR = Horizontal Rotated; ARB = Arbitrary) across all targets. Both hands were pooled for MFL and CF. Error bars denote 95% Confidence Intervals. *p′<0.05 ; **p′<0.01; ***p′<0.001; ****p′<0.0001.

Table 1 and Figure 4 show the hand movement timing data for all subjects. Unexpectedly, MFL exhibited timing differences relative to controls in the standard condition (V) for her RT and in the arbitrary condition (ARB) for her MT, suggesting that timing effects in this patient could be an unspecific tendency to be more cautious than controls before or during motor execution.

**Table 1:** Hand movement timing differences separated by hand and visual target between MFL and CF compared with the control group.

Group	Variable	Condition	Hand	Target	t-Value	Effect size
MFL	RT	V	L	L,B	>3.9*	>4.2
		V	R	T,L,B	>2.7	>2.9
		VR	L,R	R,T,L,B	>3.7*	>3.9
		H	L	R,T,B	>2.8	>2.9
		H	R	R,T,L,B	>2.8	>2.9
		HR	L,R	R,T,L,B	>3.2	>3.4
	MT	VR	L	L	3.1	3.3
		VR	R	R,T,L	>3.4	>3.6
		H	L,R	L	>2.5	>2.6
		HR	L	T	2.9	3.1
		HR	R	T,L	>2.9	>3.1
		ARB	L	L	3.6*	3.8
	CMT	V	R	L	3.5*	3.7
		VR	R	T	11.2***	11.9
		H	L	R	5.9**	6.2
		HR	L	T,B	>2.7	>2.9
CF	RT	VR	L	B	3.6*	3.9
		VR	R	L	2.6	2.7
		HR	L	R,T	>2.7	>2.9
		HR	R	R	2.7	2.9
	MT	V	L	R	2.6	2.7
		VR	L,R	R,L,B	>2.6	>2.7
		H	L	B	2.5	2.6
		H	R	R,B	>2.7	>2.9
		HR	L	L	3.1	3.3
	CMT	V	R	T	2.5	2.7
		VR	L	R	2.7	2.9
		VR	R	T	3.8*	4.1
		HR	R	R	2.6	2.8

Table 1 note: Dependent variables (RT = reaction time; MT = ballistic movement time; CMT = corrective movement time) were tested with separated modified t-tests (*p*′<0.05) for each condition (V = vertical; VR = vertical rotated; H = horizontal; HR = horizontal rotated) for each hand and each visual target (R = right; T = top; L = left; B = bottom).

* *p*′<0.01 ;

** *p*′<0.001;

*** *p*′<0.0001.

Pooled across both hands and all visual targets, MFL also displayed longer RT than the control group for all non-standard conditions (H: *t* = 3.0, *p*′<0.05, effect size = 3.1; VR and HR: *t*>5.4, *p*′<0.001, effect size>5.8). Across all visual targets, CF displayed slower RT than the control group only when using his left (affected) hand during HR (*t* = 2.6, *p*′<0.05, effect size = 2.8). Both MFL and CF revealed an overall deficit (both hands, all visual targets) of MT, relative to the control group, for VR (MFL: *t* = 2.8, *p* ′<0.05, effect size = 3.0; CF: *t* = 3.6, *p*′<0.01, effect size = 3.8). In addition, both MFL and CF took longer to correct their movements (CMT) compared to the control group during VR while using their right (unaffected) hands, across all visual targets (MFL: *t* = 2.8, *p* ′<0.05, effect size = 3.0; CF: *t* = 2.4, *p*′<0.05, effect size = 2.6). MFL also displayed an increase in CMT in condition HR across hand and target (*t* = 2.6, *p*′<0.05, effect size = 2.8).

In summary, decoupling the spatial location of the foveally-acquired visual target and the hand motion required to reach that target led to a slowing of preparation, initial movement execution, and online movement correction in these OA patients, independent of the target and with no consistent hand effect (see Table 1).

### Hand endpoints

#### Control group

Within-group analyses were conducted on the control group for hand position following the initial ballistic movement, however, no differences in endpoint accuracy (CE) or precision (VE) were observed (*p*>0.05). The controls only made 180° hand direction reversals (i.e. did not implement non-standard rule) during the conditions involving a visuomotor rotation (VR/HR; ANOVA, *F*_2,11_ = 5.2, *p*<0.05).

#### OA patients versus control group

**Figure 5:**
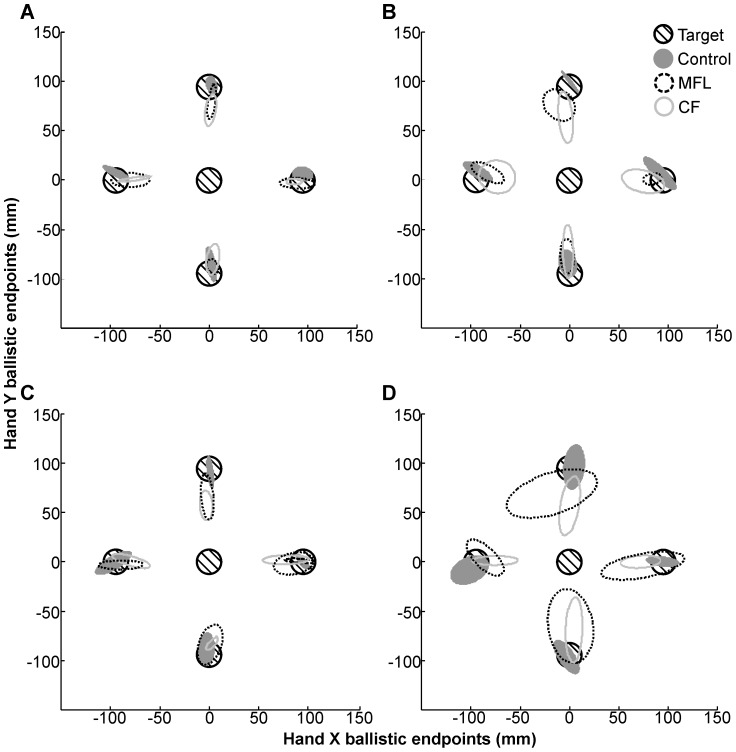
Individual hand endpoint ellipses for MFL, CF, and a typical control subject. Hand movement endpoints to four peripheral targets from the home target in (A) Vertical (B) Vertical Rotated (C) Horizontal (D) Horizontal Rotated. Both hands were pooled for MFL and CF. Open and filled ellipses represent 95% confidence intervals for patients and a typical control, respectively. Circles with cross-hatching represent starting and ending target location. Note that the systematic undershoot seen in both patients is not seen in the horizontal conditions in the + y direction towards the monitor.

**Figure 6:**
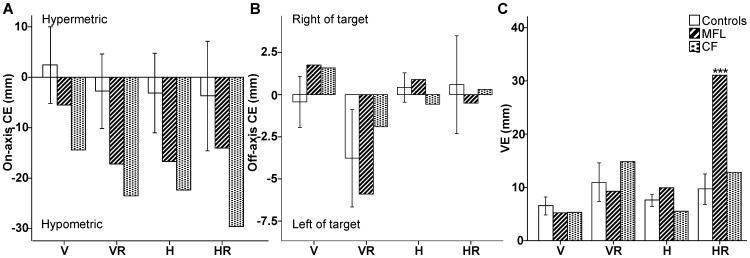
Ballistic hand endpoint data for MFL, CF, and the control group. Hand movement (A) on-axis constant error (B) off-axis constant error (C) variable error (in mm) for four conditions (V = Vertical; VR = Vertical Rotated; H = Horizontal; HR = Horizontal Rotated) across all targets. Both hands were pooled for MFL and CF. Error bars denote 95% Confidence Intervals. ***p′<0.001.

Both OA patients displayed a systematic undershoot (i.e. negative on-axis CE) of the targets in non-standard visuomotor conditions (Figs. 5B,C,D & 6A). This finding was accompanied by relatively very little direction error (i.e. off-axis CE; Fig. 6B). Indeed, neither OA patient displayed a hand movement bias towards the computer monitor in those conditions in which the hand was moving in a horizontal spatial plane while viewing the target on a vertical monitor (H/HR; see Figs. 5C,D). 180° hand movement direction reversals were observed in patients during visuomotor rotation conditions (VR and HR), as in control subjects, but significantly more than the control group for MFL with her right (unaffected, non-dominant) hand, and for CF when required to move into his affected (left) visual field (right visual target; see Table 2 for details). CF was also more variable (VE) than controls during visuomotor rotations (VR and HR) when using his left (affected) hand and when right visual targets were presented, a situation cumulating hand and field effects. CF also produced hypometric reaching errors (on-axis CE) significantly higher than controls in all non-standard conditions (H, VR and HR), but they were observed with both hands and only when the top target was presented. For MFL, VE was also higher than controls overall in HR (Fig. 6C), with the left (affected) hand when the right visual target was presented in H, and with the right (unaffected) hand when the left visual target was presented in VR. In summary, differences in hand endpoints parameters between OA patients and controls were observed only in non-standard visuomotor conditions, with no systematic hand or visual field biases across the conditions.

**Table 2:** Hand movement endpoint and error differences between MFL and CF compared with the control group.

Group	Variable	Condition	Hand	Target	t-Value	Effect size
MFL	On-axis CE	VR	R	T	−2.6	−2.7
	Off-axis CE	VR	R	R	−2.6	−2.8
		HR	R	T	−3.2	−3.5
	VE	VR	R	L	5.8**	6.2
		H	L	R	7.2**	7.6
		HR	R	R,T,L,B	>7.1**	>7.5
	DR 180°	VR	R	R	2.9	3.1
		HR	R	R,T,L,B	>2.9	>3.1
CF	On-axis CE	VR	R	T	−2.5	−2.7
		H	L,R	T	<3.6*	<3.8
		HR	L,R	T	<4.2*	<4.5
	VE	VR	L	R	5.6**	6.0
		HR	L	R,B	>2.6	2.8
	DR 180°	VR	L,R	R	>2.9	>3.1
		HR	L	R	7.0**	7.4

Table 2 note: Dependent variables (CE = constant error; VE = variable error; DR 180° =  direction reversals in the opposite direction) were tested with separated modified t-tests (*p*′<0.05) for each condition (V = vertical; VR = vertical rotated; H = horizontal; HR = horizontal rotated) for each hand and each visual target (R = right; T = top; L = left; B = bottom).

* *p*′<0.01;

** *p*′<0.001.

### Eye movement errors

**Figure 7:**
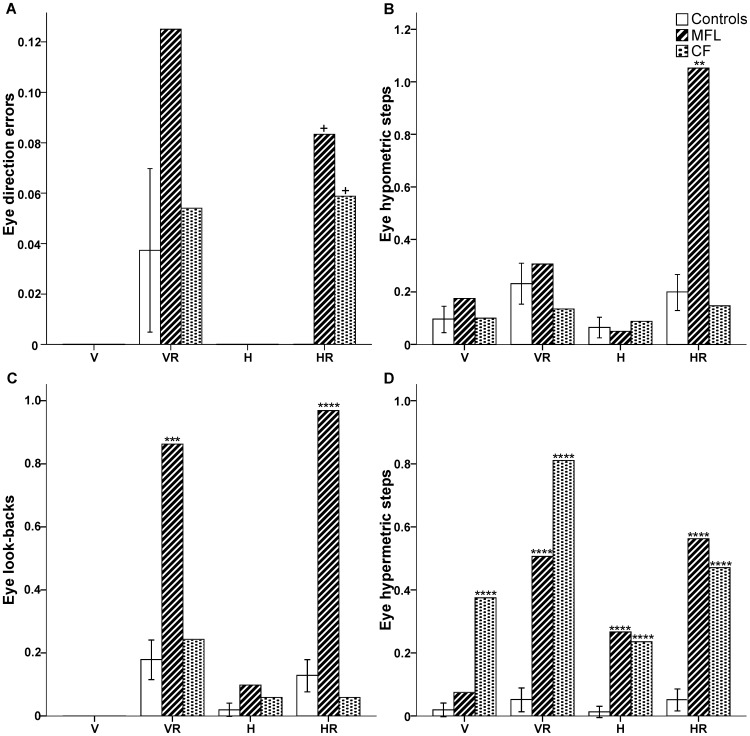
Mean eye errors performed by MFL, CF, and the control group. (A) Eye direction errors (B) hypometric steps (C) look-backs (D) hypermetric steps that have been normalized as a ratio per trial across all targets for four conditions (V = Vertical; VR = Vertical Rotated; H = Horizontal; HR = Horizontal Rotated). Both hands were pooled for MFL and CF. Note an increase in oculomotor errors for both MFL and CF during the conditions with rotated visual feedback (VR/HR). Error bars denote 95% Confidence Intervals. + No statistical comparison between the case and the control group could be performed because the control group had a mean and variance of zero. **p′<0.01; ***p′<0.001; ****p′<0.0001.

Although the hand data for the OA patients demonstrated impaired performance during the initial ballistic phase of non-standard, decoupled movements, they eventually did complete all trials within the given time limit (eight seconds). The reason for their overall success becomes clear when looking at the eye movement data. Although all subjects were instructed to foveally acquire the target, several oculomotor errors were observed in the OA patients (see Fig. 7).

#### Control group

For the most part, the control group followed the given instructions and spontaneously kept their eyes on the peripherally cued (presented) visual target. One exception was a condition main effect for the number of look-backs (ANOVA, *F*_2,12_ = 16.7, *p*<0.0001), whereby controls performed significantly more look-backs towards their hand position during the rotated conditions (VR/HR) relative to V and H (*p<*0.05).

#### OA patients versus control group

Both OA patients performed more oculomotor errors than the control participants (Fig. 7; for specific hand and target details, see Table 3). During the performance of HR, both MFL and CF performed initial saccades towards the goal location of the upcoming hand movement (eye directional errors), while none of the control subjects performed such errors (no statistical comparisons could be made, see Methods).

**Table 3:** Eye movement error differences between MFL and CF compared with the control group.

Group	Variable	Condition	Hand	Target	t-Value	Effect size
MFL	DE	VR	L,R	R	>4.1*	>4.4
		HR	R	R,T	+	+
	Look-backs	VR	L	R,B	>4.9*	>5.2
		VR	R	R,T,L,B	>2.6	>2.7
		H	L	L	3.3	3.5
		H	R	T,B	>4.2*	>4.4
		HR	L	T,L,B	>2.9	<3.1
		HR	R	R,T,L,B	>4.1*	>4.4
	Hypo-steps	V	L	B	4.2*	4.5
		HR	L	R,T,B	>4.3*	>4.6
		HR	R	T,L,B	>2.9	>3.1
	Hyper-steps	V	R	T	18.83***	20.0
		VR	L	R,L,B	>3.5*	>3.7
		VR	R	R,T,L,B	>3.7*	>3.9
		H	L	L,B	>7.6**	>8.1
		H	R	R,L	>7.7**	>8.1
		HR	L	T,B	>7.1**	>7.6
		HR	R	R,L,B	>13.2***	>14.0
CF	DE	HR	R	T	+	+
	Look-backs	VR	R	R	2.7	2.9
		H	R	T	3.2	3.4
		HR	L	R,T	>3.2	>3.4
		HR	R	T	2.5	2.7
	Hypo-steps	H	R	R	4.0*	4.3
		HR	R	T	4.3*	4.6
	Hyper-steps	V	L	R,T,L,B	>7.4**	>7.9
		V	R	R,L	>5.8**	>6.2
		VR	L,R	R,T,L,B	>2.7	>2.9
		H	L	R,T,L	>2.7	>2.9
		H	R	R,T,L,B	>7.4**	>8.1
		HR	L	R,T,L,B	>3.1	>3.2
		HR	R	R,L,B	>5.3*	>5.6

Table 2 note: Dependent variables (DE = initial direction error; Look-backs = look-backs to hand or cursor; Hypo-steps = hypometric saccadic steps; Hyper-steps = hypermetric saccadic steps) were tested with separated modified t-tests (*p*′<0.05) for each condition (V = vertical; VR = vertical rotated; H = horizontal; HR = horizontal rotated) for each hand and each visual target (R = right; T = top; L = left; B = bottom). + No statistical comparison between the case and the control group could be performed because the control group had a mean and variance of zero.

* *p*′<0.01;

** *p*′<0.001;

*** *p*′<0.0001.

MFL relied on additional hypometric steps with either hand and across all visual targets than the controls did during HR (*t* = 4.6, *p*′<0.001, effect size = 4.9). CF relied on hypometric steps only while using his right (unaffected) hand towards the right visual target during H and the top visual target during HR (Table 3).

MFL performed more overall “look-backs” towards her hand in VR (either hand, all visual targets; *t* = 5.9, *p*′<0.001, effect size = 6.2) and towards the cursor in HR (*t* = 9.4, *p*′<0.0001, effect size = 10.0) than control subjects did. CF did perform a greater number of look-backs during all the decoupled conditions (VR, H, HR) when orienting the cursor towards the top or the right visual target (Table 3).

Lastly, both MFL and CF performed more “hypermetric steps” than the control group towards the frame of the computer monitor during the decoupled conditions (VR, H, HR; *t*>8.6, *p*′<0.0001, effect size>9.1), CF already performing more hypermetric steps during direct visuomotor control (V; *t* = 10.9, *p*′<0.0001, effect size = 11.5).

In summary, both patients made more eye-movement errors compared to control subjects, particularly during the execution of decoupled visuomotor tasks, with no systematic hand or visual field biases across the conditions (Table 3).

## Discussion

The alterations in eye-hand coordination observed in the present experiment suggest a critical role for caudal SPL in non-standard visually-guided reaching, i.e. when gaze and hand direction are decoupled. The patients' hand endpoints revealed no directional errors but increased variable errors and hypometric errors during non-standard conditions in several specific comparisons with controls. In addition, unlike controls, the OA patients performed many eye movements during non-standard conditions, both exhibiting a frequent number of hypermetric step errors compared to control subjects and eye movement reversals during visuomotor rotations.

Overall, in both patients, we found no obvious and systematic differences in reaching or eye-movement parameters as a function of which hand was used, which target was reached or which direction the movement was guided. Since we found no consistent lateralised deficits, the directional hypothesis, based on allocentric directional coding, can be discarded. Instead, we suggest that the deficits seen in these unilateral OA patients reflect a global deficit in the initial decoupling and online monitoring of non-standard visually-guided reaches. The monitoring of peripheral vision involves covert spatial attention, and SPL has been shown to be integral for such covert attention shifts [4], [46]–[49]. Without an intact SPL, patients with optic ataxia may have lost their ability to attend to and represent extrafoveal ***goal and hand*** locations [4], [13]. Along this ‘positional’ hypothesis (developed by [38]), a unilateral optic ataxic patient with field dependent misreaching (field effect) would fail in all conditions decoupling hand and eye, especially if the eye does not remain still on the target. Indeed, if the eyes gaze the ongoing hand to an extrafoveal location, current hand position may be well represented but the intended target may not; conversely, if the eyes gaze the target, the intended goal may be well represented but the current hand position may not. In the one case, the impaired visuomotor system knows where the hand is, but not where to go; in the other, it knows where to direct the hand to, but not where from. In either case, the smooth visuomotor guidance will fail [38].

### The involvement of caudal superior parietal lobule in strategic control?

Incorporating a cognitive rule into a visuomotor task can lead to slower visuomotor control. The increased time required for processing an appropriate motor plan (i.e. motor strategy) for an upcoming peripherally-guided movement has been previously shown as a successful means of eliminating the ballistic visuomotor control deficits seen in OA patients [50]–[52]. In the present study we demonstrate preserved strategic control in the OA patients [53], based on their successful performance during the arbitrary mapping task relative to controls. Arbitrary visuomotor transformations have been shown to involve the integration of ventrolateral prefrontal inputs into rostral PMd [54]. While a recent study has found evidence for the involvement of foci within the PPC in processing arbitrary mappings [55], in the present study neither OA patient had difficulty preparing for them. Overall, these data imply that an intact caudal SPL is not imperative for the successful completion of cognitive-motor integration in arbitrary situations.

In addition, the OA patients were able to learn the cognitive rules of the 180° feedback rotation, although their performance did not fully match that of controls. Previous work has suggested that 180° feedback rotation tasks require cognitive-rule integration rather than more implicit mental rotation required for other amounts of feedback rotation (e.g. 60°) [5]. In addition, both OA patients in the current study were able to utilize the horizontal touch screen as a tool to guide a cursor toward the visual target on the vertical plane. Taken together, our findings suggest that the capacity to learn the appropriate rules in order to compute different levels of non-standard visuomotor transformations is preserved in OA. In contrast, the required implicit realignment of visual and proprioceptive discrepancies (i.e. sensorimotor recalibration) during decoupled visually-guided reaching appears to have been compromised. Despite intact strategic control, the increased reliance on proprioceptive inputs during decoupled visually-guided reaching [37], [56], [57] suggests that the deficits seen in these unilateral OA patients are indeed a result of impaired sensorimotor recalibration.

### Visuomotor rotation versus spatial plane dissociation

One main finding was that unilateral OA patients did not reach towards the actual direction of gaze when the gaze and reach target were decoupled by virtue of being in different spatial planes (gaze on vertical monitor, hand moving over horizontal table). Rather, their reaching bias occurred in the plane that the hand was moving in. This finding confirms our previous demonstration of this preserved behaviour in a bilateral OA patient [4]. In a similar situation, Alzheimer Disease (AD) patients were not able to accommodate such spatial plane differences, instead producing hand movements that were towards the physical location of the viewed monitor [58]. We have proposed previously that AD patients may be experiencing a disconnection between prefrontal and parietal areas, areas whose connectivity is likely important for cognitive-motor integration [59]–[61]. The current study's findings that OA patients have no specific trouble when dissociating the plane of eye and hand movements suggest that an intact, independent neural pathway is used in such condition, potentially the left dorsolateral parieto-frontal network [18] that is involved in choosing the appropriate distal limb orientation for purposeful tool-use, or the same integration of ventrolateral prefrontal inputs into rostral PMd [54] as involved in arbitrary visuomotor transformations.

For OA patients, visuomotor rotation led more often than plane dissociation to pathological behaviour. It may be because plane dissociation simply requires transposing a motor plan to another location within the same hemifield, whereas inverting the direction of eye and hand motion may involve a transfer towards or away from the unilateral patient's damaged hemisphere (for the left and right targets). Alternatively, visuomotor rotations result in a larger dissociation between proprioception and vision. In addition to the dissociation between peripheral vision of the hand and foveal vision of the cursor being moved to the target, the hand also has to be guided in a direction opposite to the cursor. Whether this is due to the demands of computing an inverted difference vector [62], the greater inhibition requirements in these conflicting situations [21], [63], [64] or a more extensive network for ‘anti-movement’ versus ‘postural adjustment’ type tasks, remains an open question.

### Oculomotor errors during non-standard reaching in optic ataxia

The second main finding in the present experiment is that the OA patients were unable to simply look at a target and then reach or guide a cursor to that target as instructed. Rather, they made a number of eye-movement errors which allowed them to ultimately complete the trials. We believe that these errors reflect oculomotor strategies that these patients have developed in order to successfully interact with the external world, particularly in situations in which gaze and reach direction are decoupled. We propose that the most parsimonious explanation for the eye-movement behaviors observed in these patients is that 1) they serve to assist in locating the upcoming spatial location of the goal of the hand movement (i.e. priming that location), and 2) they serve to update the difference vector between the current location of the hand and the goal of the movement.

The OA patients performed the greatest number of saccadic errors during the performance of both tasks involving a visuomotor rotation (VR/HR). These eye-movements likely served to prime the remembered location of the upcoming goal requirement (cursor to the target). This behaviour has been previously shown during a series of object manipulation tasks [65], [66]. The authors of these studies proposed that a series of eye movements towards the edges of an object about to manipulated, the upcoming target, and the end-goal of the movement often preceded the hand movement in order to successfully predict the spatial location and timing of the upcoming hand movements. In the present context, during the performance of HR, both OA patients utilized initial saccadic direction errors towards the transformed (cursor) location of the upcoming hand movement direction, something that none of the control participants did.

We also suggest that these OA patients are often updating the difference vector between their eye and hand using vision. Previous work using transcranial magnetic stimulation suggests that the dorsolateral PPC may be crucial for maintaining a difference vector between the current hand location and the desired movement goal [67]. In the present study, support for this idea comes from the look-backs and the hypermetric steps performed by the OA patients. These additional eye movements may provide a means to re-couple the natural linkage between eye and hand movements [37], [42], [68]–[70]. Overall, the OA patients performed the most hypermetric steps during the decoupled visually-guided reaches. The additional hypermetric saccades were most likely performed by the OA patients in order to utilize an additional cue within the environment (to replace the peripherally-viewed hand) in order to complete the task. The increase in oculomotor errors performed by CF towards the end of the movement may reflect the online control deficits seen previously during target jump paradigms [36]. We suggest that the additional saccades performed by the OA patients may serve to foveally update the relative position of the end-effector (hand/cursor) and the visual target in order to recalibrate the hand movement goal.

Overall, these scan-path data reiterate the role of an intact caudal SPL in simultaneously representing and integrating proprioceptive (intrinsic) and visual (extrinsic) information for successful planning of visually-guided reaching [71], especially as the eye and the hand movements become spatially decoupled [37], [56], [72].

### Hypometric reaching in optic ataxia

Hypometric reaching deficits in extra-foveal reaching seen in primates with caudal SPL damage (for example, [73], [74]) may be partially explained by a role of this region in covert attention changes [46], [49] between eye and goal locations. As well, the reported gaze-biased undershooting of extra-foveal targets could result from an increased reliance on coding of the decoupled reach and gaze directions in intrinsic (limb postural) coordinates [56], [72]. Without the benefits of overt visual updating of limb position [65], [75]–[77], decoupled reaching deficits seen in OA patients may reflect difficulty with the conversion from the eye-centered (extrinsic) coordinates of the visual goal [77]–[84] into the limb-centered (intrinsic) coordinates needed to guide the decoupled limb [37], [56], [72], [77]. Previously, it was thought that a limb-centered reference frame is only required later in the movement correction phase [81].

Similar to previous reports [2], [35], in the present study, the OA patients did not display initial hypometric reaching during direct visuomotor control (i.e. standard condition in free vision). Both OA patients did, however, undershoot their hand during the decoupled visually-guided reaches relative to the standard condition (negative on-axis CE; Figs. 5 & 6A). In contrast to previous work on unilateral OA patients utilizing central fixation paradigms [35], [36], no obvious misreaching to the contralateral visual field (field effect) or by the affected hand (hand effect) were observed. It may be that testing left handed and ambidextrous patients may reduce laterality in eye-hand coordination, however previous work with these patients [13], [22], [36], [44] suggests that this is not the case. Rather, not preventing direct (foveal) vision of the target eliminated the visual field effect (as predicted) and the hand effect in these patients. Note that the hand effect is reduced when the hand is calibrated by vision at the start and movement is performed in lighting conditions [13]. Overall, the OA patients appear to display a global motor deficit when relying on decoupled proprioceptive and visual inputs when they are able to foveate the visual target.

## Conclusions

The results of this study suggest that the right dorsal stream missing in both of the OA patients that we studied (i.e. caudal SPL and its direct connection to rostral PMd) is a critical component of the global network involved in overcoming the natural coupling of eye and hand movements. Despite preserved strategic control, we suggest that an intact caudal SPL is crucial for maintaining and updating hand location in peripheral vision in situations requiring decoupled eye-hand coordination.
